# Homœopathy, Its Principles and Practice

**Published:** 1855-08

**Authors:** C. A. Hathwell

**Affiliations:** Virginia, Cass Co., Ill.


ARTICLE V.—*Homœopathy, its Principles and Practice.* By C. A. Hathwell, M. D., Virginia, Cass Co., Ill.

——If any, speak, for him have I offended.
*Brutus.*
——I came to bury Cæsar, not to praise him.
*Mark Antony.*

We are given to understand through the medium of various journals, that the Legislature of Michigan, in their wise deliberations, have concluded on having a Chair of Homœopathy estabtablished in the Medical Department of their State University, at Ann Arbor.

Now, as Homœopathy is truly one of the novelties of the age we live in, we propose to say a few words about this system, in which many are at this very time so steadfastly and perseveringly pursuing; but, however, neither particularly to advocate or condemn it, for though we know a little about its principle or theory, we honestly confess knowing still less about its practice.

Homœopathy is defined "similar disease," which, in its turn, means that the remedy is the same as the cause of the disease.

This is the discovery of Dr. Hahnemann, the founder of Homœopathy, and dates, we believe, from 1790—the epoch of the French Revolution,—verily, an epoch of novelties.

For example: in a healthy person coffee produces excitement, in a sick one it removes it; in a healthy person opium produces sleep, in a sick one wakefulness. It is on this principle that in persons whose vitality has been suspended by exposure to cold, animation is restored by rubbing them with snow; and that burns are most efficaciously treated by heat, either by direct exposure to fire, or by partially exciting heat.

Now, this is Homœopathy in its original and etymological sense, but not the sense in which it is generally known to the public. This latter sense has reference to another discovery of Dr. Hahnemann, called *the development of power in medicinal substances.*

By daily experience he found that physic applied in gross form, as under the old (or Allopathic) system, acted much too strongly; he, therefore, diluted it with water or Spirits of Wine, and thus discovered that its efficacy was increased in proportion to its weakness, the less matter, the more spirit. For instance: the medicine is prepared in the following manner. Take one drop of strong tincture, say tincture of Rhubarb, mix it with ninety-nine drops of Spirits of Wine,—that makes the first power. Take one drop of that diluted tincture and mix it with ninety-nine drops of Spirits of Wine, and you have the second power. Take one drop of the second power and mix it with ninety-nine drops of Spirits of Wine, and then you have the third power of Rhubarb, and so on till you come to the thirtieth power, and this is the first power that can be used efficaciously; it is the lowest Homœopathic power that is used. The power in common use, a pill or globule of the thirtieth power, is said to contain one decillionth part of a drop of strong tincture,—a decillion is expressed on the Continent by 34 figures, in England by 61. Homœopathic medicine is also prepared in a dry state by trituration, which is said to magnetise it, or increase its power.

By following up this same system with perseverance for months, or years, you raise the power, that is, weaken the material solution of the medicine to any extent you please.

Jeninchen, a patient and most persevering German Homœopathist, of Wiemar, whom we believe is now deceased, raised the powers of Homœopathic remedies by weakening them, to a greater extent than any other that we have heard of. Arsenic, for instance, he raised to the forty thousandth power. He must consequently have taken the drop of tincture forty thousand times from one vial, and mixed it forty thousand times with the Spirits of wine in another vial, and made a memorandum of it at the same time. Moreover, the vials must be particularly clean, rinsed a dozen times, and the water not only wiped but evaporated from them by heat. The trouble and the time, therefore, are very great, and consequently these high powers which contain as near as possible nothing of medicine, are very expensive, the preparation of them being the labor of years. The effects of these high powers of Jeninchen's are said to be bordering on the wonderful! By some means, only known to him, he increased the effect to a degree never before obtained in powers prepared by any one, or by machinery!!

To complete the preparation of the medicine, a number of pills of so diminutive a size that some ten or twelve of them would not more than occupy the capacity of a mouse's ear, composed of Sugar of Milk, are made by the confectioner, and dipped in the diluted tincture. These sugar of milk pills constitute the homœopathic medicine, and even these are not taken entire always, for two, three or five (Lutze always uses five) are put into half a tumbler of water and dissolved, and you take a spoonful of the mixture once or twice a day, and this is all your treatment, except that you are placed upon a rigid diet, and forbidden the use of every thing stimulating, as wine, beer, coffee, tea, mustard, pepper, &c., nay, you are carefully enjoined to *smell* nothing that excites; even the effluvium of the pepper-box is decidedly injurious, and almost any other tooth-powder but fine charcoal will prove a counter-agent to the delicate medicinal spirit that has been introduced into your system. A young lady under homœopathic treatment called to see a female friend of hers, whom she found bleaching a straw bonnet, by exposing it to the fumes of brimstone under a barrel. She accidentally smelt the sulphur, her physician in his inquisition found it out, and with terror and consternation depicted in every feature, he exclaimed to her, "Horrible! oh, most horrible! how could you do such a thing?"

Dr. Lutze, of Cothen, (if still in existence, and we have not heard of his decease,) is, we believe, the most distinguished of living Homœopathists in Germany. His cures are said to present something like the appearance of magic. His powers are also nearly as high as those of Jeninchen's, that is twenty, thirty, and even forty thousand. There is a spirituality also about the man which invests his professional method with something of a religious character. Indeed, the tendency of homœopathy is so decidedly spiritual that it naturally leads its professors in spite of themselves into mystic and religious feelings. How is it possible for a man to escape them when he becomes convinced from experience that the less material his medicine becomes the more efficacious it proves? "The less matter the more spirit" is his admitted maxim; and if this does not tend to make a spiritualist of a man we know not what will.

Hence it comes to pass that the most distinguished and successful homœopathists instruct their patients to look to God for a blessing upon the medicines, for without His aid all material means must prove in vain.

Lutze, we are told, carries the devotional spirit into the sick chamber, and is himself so deeply imbued with the religious feeling and the high religious mission of the medical profession, that it is more than probable that this medicinal spirit in himself is much more efficacious than that imprisoned spirit in the forty thousandth power of his diluted sugar of milk pills.

Indeed, it has been observed by his disciples, that instead of following to the letter the rules of homœopathic treatment, he transgresses them frequently with success. After hearing the symptoms of a complaint, he put his hand on a medicine and took it out; a pupil observed that "that was wrong." "Never mind," said Lutze, "it will do;" and *do it did!*

Wonderful stories are told of homœopathists. We shall give one of Dr. Hahnemann, the founder of the system.

"This eminent physician had invited a small company to his house, and while all were in the full enjoyment of mirth, a young lady, the daughter of a physician, was observed by Dr. Hahnemann seated in a corner, suffering from a most violent toothache. Hahnemann said to her, "Well, Emma, my dear, has not your father prescribed anything for your toothache?" "Yes," she replied, "he did, but all to no purpose; be kind enough to administer something to me."

*Hahnemann.*—Provided your father do not take it amiss.

*Emma.*—He shall know nothing of the matter if it does me good."

"Dr. Hahnemann drew from his pocket a small vial containing a few globules, and requested the young lady to smell it once. The pain immediately increased to an almost insupportable degree, but within a quarter of an hour had wholly ceased. Emma deemed herself very happy. She requested Dr. Hahnemann to give her the vial, which he readily assented to, but with the following injunction: "You must promise me not to smell it except on a return of the pain; if you should do so the pain will instantly come back, and the remedy will then be of no avail." Emma promised to obey the Doctor's precaution, and went home with the vial, merry and happy. Several months elapsed without a return of the toothache. In the meantime, Emma was engaged to a young physician, and one day in the presence of her intended her father said to her, "well, Emma, where's your toothache?" Emma looked considerably embarrassed at the question, and being warmly solicited to disclose the reason, related what had passed between Hahnemann and herself. Father and lover both burst into a hearty laugh. Emma by her father's request then produced the miraculous vial; both smelt it in derision, and asked Emma to smell it also. She however declined, stating that Dr. H. had strictly forbidden her to do so, as the pain would promptly return. They again pressed her, assuring her it was harmless, and yielding to their importunate entreaties she smelt it, and immediately after was seized with a return of the toothache in its most agonizing form. Her father and the young physician were struck with astonishment, and grieved at such an extraordinary result. Emma quickly sought Dr. Hahnemann in order to make full confession to him. After having found the Doctor, and divulging the facts, he accosted her "more in sorrow than in anger," "Emma, Emma, you have indeed been disobedient, and you have paid the penalty." With these words he withdrew, but soon returned, and producing another vial requested her to smell it; she complied, and the pain was at once assuaged. Having witnessed such an extraordinary phenomenon, the father and the future husband were converts to the new doctrine, and became eminent homœopathists."

It is asserted that Lutze produces similar marvelous results. Boninghansen, of Munster, is more material and less successful; but he stands next to Lutze, it is said in popular estimation. Homœopathy has made great progress in Germany, and many other parts of the continent, we there find homœopathic families and boarding houses, where patients are kept in condition for physicians.

Homœopathists have observed different effects and results in different localities. For instance, in England, and on the continent, in London some ascribe it to diet—the heavy beverage, port, sherry, porter, and ale, which with tea and coffee are such powerful counter agents as render homœopathic medicines of little or no use. The system originated in Germany, its discoveries were German, and it is very probable that its treatment requires modifications, adapted for every variety of people and climate.

Of its *worth*, we shall say but little; let its followers and believers pursue the "even tenor of their way," but we must make a few remarks upon its spiritual or immaterial character. "The less matter the more spirit," is the leading principle of homœopathy. "Give as little medicine as possible—the minimum quantity is the most efficacious, it is the spirit that cureth, and spirit is not to be measured in value, by the amount of the body that contains it A violent remedy for a human body, is like a violent remedy for a political body; it is a revolution—a convulsion—and as political experience has proved the one to be ineffective, so human experience has found that the violent *Allopathic* medicines of the common drug shops are insufficient to diminish the amount of human suffering. In both cases a more spiritual, deep and recondite remedy is indispensable."

Such is the reasoning of Homœopathy, or immaterial medicine, and such the practice, of which the assembled wisdom of the State of Michigan desire to have an established chair in their State University.

Should this article meet an Homœopathic eye, the reader will perceive we have applied to ourself, the name of Allopathic, which has been bestowed upon us, but why I am unable to account for. Who, and what are allopathic physicians? What is meant by that title, and what is their particular practice? Will some homœopathist explain? We know of none to whom this name is applicable; certainly not to the regular profession of medicine, although many of them without straining, have gulped the title down. For ourself, we maintain the ground, that the term is a misnomer, wholly inappropriate to the scientific practitioner, but as we penned this article to exhibit the reasoning and theory of homœopathy, and not in defence of our system or ourself, we shall forbear further observations as superfluous.

Should this article *not* come in contact with an homœopathic eye, I can again quote the words of Brutus, and say—

"Then none have I offended."

